# Virtual Open House: Incorporating Support Persons into the Residency Community

**DOI:** 10.5811/westjem.2022.10.57468

**Published:** 2022-12-21

**Authors:** Hurnan Vongsachang, Aarti Jain

**Affiliations:** Los Angeles County + University of Southern California Medical Center, Department of Emergency Medicine, Los Angeles, California

## BACKGROUND

Medical trainees rely on supportive personal relationships when navigating the challenges encountered during residency training.^1^ Unfortunately, the demands of the training environment may erode these pre-existing relationships and negatively impact residents’ psychological wellbeing. 1 Further, the geographic separation inherent to the residency match process and the recent coronavirus 2019 (COVID-19) pandemic may limit in-person support from friends and family members.

A qualitative study conducted at our institution suggested that an additional barrier to effective communication between residents and their support persons (SP) is dissonance between residents’ personal and professional identities.^2^ Residents encounter challenges discussing workplace stressors with individuals who have minimal understanding of the clinical environment.^1^,^2^ Study authors recommended that future wellness interventions attempt to reduce this dissonance by proactively providing SPs with greater insight into the residency training experience.^2^ This recommendation is in line with existing literature highlighting the importance of social relatedness—engaging in meaningful conversation and feeling understood—in enhancing psychological well-being. 3,4 In light of this, we created an innovation aimed at familiarizing residents’ SPs with the residency experience. To our knowledge, there are limited existing formal interventions with this objective. 1,5–7

## OBJECTIVES

We developed a virtual open house (VOH) to invite residents’ self-selected SPs into the residency community and provide them with greater insight into the clinical and non-clinical training environment.

## CURRICULAR DESIGN

All 72 postgraduate year PGY1-4 residents in our emergency medicine (EM) residency training program and their self-selected SPs received an electronic invitation to attend a two-hour VOH in April 2021. The VOH was scheduled during protected educational conference time due to prior studies suggesting that wellness interventions are perceived as a time burden and are limited by a lack of available time. 8–10 The SPs were self-selected by residents and were not limited to any population or geographic location.

As the aim of the VOH was to create an opportunity for residents to share details of their training environment, content selection was shaped largely by resident input. A VOH Task Force, created and led by HV, consisted of a select group of residents representing each postgraduate training year as well as individual resident leads of extracurricular committees. Session content, structure, and format were informed by Kern’s six-step approach to curriculum development^11^ and guided by a faculty member (AJ), given her medical education fellowship training, master’s degree in academic medicine, and experience in residency program leadership.

During the VOH, the residency program director first provided an overview of the impact of COVID-19 on the resident training experience and oriented guests to the broader hospital system. After this introduction, resident representatives summarized the day-to-day clinical responsibilities and non-clinical opportunities that characterize each residency training year. Additional residents then provided updates on the current projects and activities of various resident-led special interest committees (ie, social EM, global EM, resident wellness). During each of these segments, speakers included photos and videos to better familiarize attendees with the physical work environment and the specific members of the residency community. The program then concluded with an opportunity for large-group discussion and reflection among all attendees. While the virtual modality limited the use of interactive educational strategies, we incorporated a combination of formal presentation, large-group debriefing, and reflection to deliver session content.

### Methods

This study received institutional review board exemption from the University of Southern California. We conducted a mixed-method analysis of participants’ experiences attending the VOH. Immediately after the conclusion of the VOH, all residents and guest attendees received an electronic invitation to complete an anonymous survey regarding their experiences attending the VOH (Appendix 1). This survey was created by both authors (HV and AJ) and reviewed by faculty members from the emergency department’s education division for content validity.^12^

Additionally, six months after the conclusion of the VOH, we conducted two virtual focus groups with a convenience sample of SPs who had attended the VOH. Focus groups were scheduled at a delayed time point to explore any sustained perceptions or impacts of the VOH experience. Five SPs participated in each of the two focus groups, both of which were led by AJ given her experience in focus group facilitation. A semi-structured interview guide was developed by AJ and HV and aimed to explore a deeper understanding of the VOH experience as well as probe for any change in the conversational dynamic between SPs and residents following the VOH (Appendix 2). Interviews were audio-recorded, de-identified, and professionally transcribed.

We used an inductive thematic analysis approach.^13^ Understanding that our perspectives may influence interpretation of transcripts, we offer some background information: 13 Author AJ is an EM faculty member who has created departmental wellness initiatives and has experience in qualitative medical education research. HV was the EM resident lead of the residency wellness committee and has received training in qualitative research through her master’s degree in public health. We initially analyzed both transcripts and generated code definitions. After refining the coding framework, we developed thematic categories and reorganized them until consensus was achieved. Trustworthiness was enhanced by use of reflexivity, memoing, and an audit trail.

## IMPACT AND EFFECTIVENESS

### Quantitative Analysis

Of the 155 individuals who attended the VOH 60 (38.7%) were residents, 86 (55.5%) guests, and 11 (7.1%) faculty members. Forty residents (66%) and 47 guests (54.7%) responded to the post-VOH survey. Of the SPs who responded to the post-session survey, 89% were parents or other relatives. Additional demographic information for SPs is found in Appendix 3. Overall, attendees reported that they enjoyed the VOH (95% residents [38]; 98% guests [46]). Most respondents reported that the VOH helped to foster a greater sense of community (85% residents [34]; 73% guests, [34]), and the majority suggested that they felt more comfortable engaging in conversations regarding workplace challenges (77% residents [27]; 94% guests [44]).

### Qualitative Analysis

The SPs reported that participation in the VOH facilitated subsequent dialogue with their residents and provided them with an increased sense of comfort and familiarity with the residency training environment. Representative quotations are included in the Table. After participating in the VOH, SPs reported that their improved insight into the training environment offered them new “talking points” when engaging in subsequent dialogue with residents. Not only did the VOH provide additional conversational “clues,” but emboldened participants to initiate dialogue on topics that had previously been unaddressed. The SPs also hypothesized that their participation in the VOH allowed them to engage in deeper and more intimate conversations with residents than those who had not attended the session.

Participants also appreciated the opportunity to virtually “meet” their residents’ colleagues and supervisors, finally “put[ing] faces to names.” They were comforted by gaining an increased understanding of the emotional and structural support provided by the residency program and by directly witnessing the collegiality among trainees and faculty members. Several participants referenced the physical and emotional separation caused by the COVID-19 pandemic and reported that their concerns about their residents were alleviated by receiving greater insight into, and familiarity with, the residency community.

The focus groups also provided insights into areas for improvement during future iterations of the VOH. Participants requested the addition of virtual breakout rooms to provide them with opportunities for more intimate, small-group conversations. They also voiced enthusiasm for more longitudinal forms of connection to the residency community (ie, monthly newsletters or local parent support groups). Future iterations of this innovation can modify the frequency of VOH events and add in-person or hybrid activities when social distancing restrictions are lifted. Additionally, educators can consider including explicit instruction to SPs on effective support and communication techniques and incorporating didactic components on well-being and burnout. To enhance our understanding of the impact of a VOH, future studies should explore resident perceptions of changes to their perceived social support networks and the quality and quantity of dialogue with SPs after participation in a VOH.

## LIMITATIONS AND CONCLUSION

Our innovation involved a single institution and medical specialty, which may limit its generalizability. The SPs were self-selected by residents; individuals who attended and completed the post-session surveys may have been susceptible to varying levels of response bias. Additionally, as we did not establish levels of pre-existing medical knowledge among SPs, the perceived utility of the VOH in offering topics for future conversation may not be broadly applicable. Our qualitative findings are limited by the small sizes of our focus groups and the social desirability bias inherent to many focus group discussions. In addition, frank discussion from SPs regarding their VOH experience may have been inhibited by use of a member of program leadership (AJ) as the focus group facilitator.

Despite these limitations, the use of a VOH may help encourage future dialogue between residents and their SPs about the residency training experience. Residency program leaders should consider adopting interventions aimed at reducing identity dissonance by incorporating residents’ support persons into the residency community.

## Supplementary Information







## Figures and Tables

**Table:** Themes with exemplary quotations describing participants’ perceptions of the virtual open house experience.

Theme 1: Enhancing Future Dialogue Between Loved Ones and Residents
Providing content for future conversations “I think this virtual Zoom helped me know the whole program and gave me some opportunities on talking points that XXX may not have brought up, you know. Sometimes she would be so focused on only one thing because she’s busy, she’s tired. Having some other conversation about, “I saw your video” or “tell me about that person.” I think that gave me little clues to have different conversations and ask her different questions so she could share some other parts of work that she didn’t think to do.” [FG2] Promoting deeper conversations “I think having the faces and the names, it opens different doors to have a little bit more personal conversation. Not everybody from our family could attend that day, so I can see how the conversations are different when I attended and her dad couldn’t attend or her spouse couldn’t attend. I think the connectivity has definitely gone to a deeper level.” [FG1]
Theme 2: Deepening Support Persons’ Understanding of the Residency Experience
“Putting faces to names” “The other thing that I really liked about the open house was getting to see his friends, because we’re not there to see them. And I know that his companions in the XXX year are what makes it possible to do the work that he does. He’s very tight with them and they support each other really well. So I was glad to see everybody’s face come through there. That was really meaningful to us being that we live in XXX and just can’t get there as often as we’d like, especially in the COVID year and a half. And who knows how often we’ll be able to be together. Right? Outreach was so much appreciated.”[FG1] Providing insight into the emotional support provided by the residency community “It was very heartwarming, and you felt as a parent that your kid is in good hands. It doesn’t matter how old the children get, but it’s like, “okay, are they in a good place? Are they learning what they need to be learning at this point?” And med school has been hard. Training is harder, and that is known. So that part was very comforting.”[FG2] Enhancing an understanding of the training structure “I remember being encouraged and comforted that the residents helped one another. The more advanced residents helped their first years and second years and it seemed to be in a very structured way.”[FG1] Bridging the geographic separation “He doesn’t really download what’s hard. So, we try to come every couple months, but we couldn’t of course for that year and a half. And, so just not knowing how safe he was and how the mental health strain….When I’m with him, I can see how he’s doing. Just as a mother, all you parents can tell. It’s just been something to get used to, the actual physical distance and not really knowing. You can’t walk your child’s path in any case, but this one’s particularly hard, I think.” [FG1]

*FG1*, Focus Group 1; *FG2*, Focus Group 2.
