# Supplementary material for: Virtual Open House: Incorporating Support Persons into the Residency Community
Source: West J Emerg Med. 2022 Dec 21;24(1):79–82. doi: 10.5811/westjem.2022.10.57468 (PMC9897241; doi:10.5811/westjem.2022.10.57468)
Supplement: Supplementary file 1 [file wjem-24-79-s001.docx]

Appendix 1.

Post-Virtual Open House Survey for Support Persons (2021)

SECTION 1.

1. Your age:
2. Your gender:
   1. Male
   2. Female
   3. Non-binary
3. Your resident’s training year:
   1. Incoming PGY1
   2. PGY1
   3. PGY2
   4. PGY3
   5. PGY4
4. How are you affiliated with LAC+USC Emergency Medicine Residency?
   1. Parent
   2. Relative
   3. Partner
   4. Friend
   5. Other

SECTION 2.

Please rate the following statements (1 = strongly disagree, 5 = strongly agree).

1. I enjoyed the Virtual Open House.

| 1 | 2 | 3 | 4 | 5 |
| --- | --- | --- | --- | --- |

1. I feel like I am now more a part of the LAC+USC Emergency Medicine family.

| 1 | 2 | 3 | 4 | 5 |
| --- | --- | --- | --- | --- |

1. I would like to attend future Virtual Open House events for the residency.

| 1 | 2 | 3 | 4 | 5 |
| --- | --- | --- | --- | --- |

1. I now have a better understanding of my loved one’s individual experience during residency training.

| 1 | 2 | 3 | 4 | 5 |
| --- | --- | --- | --- | --- |

1. I now have a better understanding of the training and workplace environment at the LAC+USC Emergency Department.

| 1 | 2 | 3 | 4 | 5 |
| --- | --- | --- | --- | --- |

1. After the Virtual Open House, I feel more comfortable ENGAGING in conversations with my loved one about their workplace challenges.

| 1 | 2 | 3 | 4 | 5 |
| --- | --- | --- | --- | --- |

1. After the Virtual Open House, I will be more likely to INITIATE conversations about workplace challenges with my loved one.

| 1 | 2 | 3 | 4 | 5 |
| --- | --- | --- | --- | --- |

SECTION 3.

Please rate the following statements in terms of session usefulness (1 = not useful at all, 5 = extremely useful).

1. Program Director’s Welcome and Impact of COVID-19

| 1 | 2 | 3 | 4 | 5 |
| --- | --- | --- | --- | --- |

1. Graduated Responsibilities: Insider’s Scoop

| 1 | 2 | 3 | 4 | 5 |
| --- | --- | --- | --- | --- |

1. Overview of Committees

| 1 | 2 | 3 | 4 | 5 |
| --- | --- | --- | --- | --- |

1. Q&A

| 1 | 2 | 3 | 4 | 5 |
| --- | --- | --- | --- | --- |

1. Reflections Session

| 1 | 2 | 3 | 4 | 5 |
| --- | --- | --- | --- | --- |

1. Any final comments or suggestions in regards to the Virtual Open House?
