# Supplementary material for: Virtual Open House: Incorporating Support Persons into the Residency Community
Source: West J Emerg Med. 2022 Dec 21;24(1):79–82. doi: 10.5811/westjem.2022.10.57468 (PMC9897241; doi:10.5811/westjem.2022.10.57468)
Supplement: Supplementary file 2 [file wjem-24-79-s002.docx]

APPENDIX 2

Focus group interview guide

1. Tell us about your general impression of the Virtual Open House
2. What were your favorite parts of the Virtual Open House experience?
3. In what ways, if any, did your participation in the open house change your understanding of your loved one’s residency experience?
   1. PROBE: What was your understanding of their experience prior to the Virtual Open House?
4. In what ways, if any, did your participation in the open house change your feeling of connection to your loved one’s residency experience?
5. In what ways, if any, did your participation in the open house impact the likelihood that you **engage** with your loved ones in conversations regarding workplace challenges moving forward?
6. In what ways, if any, did your participation in the open house impact the likelihood that you will **initiate** conversations regarding the workplace with your loved one?
7. Moving forward, what steps do you think you will take to support your loved one during their residency training?
8. Any general feedback or suggestions?
