# Supplementary material for: Virtual Open House: Incorporating Support Persons into the Residency Community
Source: West J Emerg Med. 2022 Dec 21;24(1):79–82. doi: 10.5811/westjem.2022.10.57468 (PMC9897241; doi:10.5811/westjem.2022.10.57468)
Supplement: Supplementary file 3 [file wjem-24-79-s003.docx]

Appendix 3.
